# Effects of a myostatin mutation in Japanese quail (*Coturnix japonica*) on the physicochemical and histochemical characteristics of the *pectoralis major* muscle

**DOI:** 10.3389/fphys.2023.1172884

**Published:** 2023-03-30

**Authors:** Dong-Hwan Kim, Boin Lee, Joonbum Lee, Benjamin M. Bohrer, Young Min Choi, Kichoon Lee

**Affiliations:** ^1^ Department of Animal Sciences, The Ohio State University, Columbus, OH, United States; ^2^ Department of Animal Science and Biotechnology, Kyungpook National University, Sangju, Republic of Korea

**Keywords:** myostatin mutation, meat quality, muscle fiber conversion, pectoralis major muscle, quail

## Abstract

The aim of this study was to compare the carcass, meat quality, and histochemical characteristics of *pectoralis major* (PM) muscle between wild type (WT) and myostatin (Mstn) homozygous mutant (HO) quail lines. The HO quail line exhibited significantly heavier body weight (HO vs. WT, 115.7 g vs. 106.2 g, approximately 110%) and PM muscle weight (HO vs. WT, 18.0 g vs. 15.2 g, approximately 120%) compared to the WT (*p* < 0.001). However, the two groups had similar traits (pH, redness, yellowness, and drip loss) for meat quality, although slightly higher lightness and cooking loss were observed in the mutant quail (103% and 141%, respectively, *p* < 0.05). For histochemical traits of PM muscle, Mstn mutant quail exhibited lower type IIA and higher type IIB percentage in the deep region than WT quail (*p* < 0.05), indicating a fiber conversion from the type IIA to IIB. However, the two quail lines had comparable histochemical traits in the superficial region (*p* > 0.05). These data suggest that Mstn mutation greatly increases muscle mass without significantly affecting meat quality.

## 1 Introduction

In the past several decades, the consumption of poultry meat and development of related industries has been steadily growing due to increasing consumer preference for poultry meat (Soglia et al., 2016). This progressive growth is primarily related to the perception of an improved nutritional profile of poultry *versus* meat from other livestock species, such as the low-fat content and the high quantity of high quality protein found in lean poultry meat (Soglia et al., 2016). Meat-type poultry including chicken and turkey have been selected for heavy body and breast weights over multiple generations, and generally exhibit increased muscle mass and faster growth rate when compared with previous generations (Nestor et al., 2008; Putman et al., 2017). However, with the fast-growing performance of modern poultry, excessive fat deposition is one of the biggest concerns for producers and processors, which can lead to decreased consumer acceptability and economic loss (Fouad and El-Senousey, 2014). Thus, decreasing fat accumulation with high rates of lean growth would be a goal in the poultry industry. (Fouad and El-Senousey, 2014).

Myostatin (Mstn) is a well-studied gene that regulates muscle mass and fat deposition. In fact, mutation or knock-out for this gene has been documented to increase muscle mass and decrease fat content in different animal species, such as pigs, cattle, and mice (McPherron and Lee, 2002; Fiems, 2012; Cai et al., 2017). Our previous study also reported that the Mstn knock-out quail line exhibited approximately 30% lower body fat content and approximately 20% heavier muscle mass with increased muscle fiber numbers compared to the wild type (WT) quail line (Lee et al., 2020). Thus, the Mstn gene can be considered as an economically important gene, and genetic selection or manipulation of this gene can contribute to developing leaner lines of poultry that could increase consumers and producers satisfaction.

It has been shown that muscle fiber composition can influence meat quality due to differences in contractile and metabolic traits of muscle fiber types, especially in pigs and poultry that exhibit a rapid rate of *postmortem* metabolism (Choi and Kim, 2009). As the proportion of large diameter glycolytic muscle fibers increases, there is less space between the muscle fibers particularly when compared with small diameter oxidative muscle fibers (Petracci et al., 2017). After exsanguination, limiting the space available to the capillaries that normally remove lactate from the muscle leads to accumulation of lactic acid, consequently causing a more rapid pH decline in muscle, and thus poor meat quality (Petracci et al., 2017). Disruption of Mstn can switch fiber type from slow-to fast-twitch fibers in mature resting muscles in various species of animals, including mice, pigs, and cattle (Stavaux et al., 1994; Hennebry et al., 2009; Qian et al., 2015). However, to our knowledge, there are no studies that have reported both the muscle fiber type composition and meat quality characteristics in any poultry species. Although weights of chicken breast muscle having only type IIB myofibers were not significantly increased by Mstn mutation (Kim et al., 2022), weights of quail breast muscle containing both type IIA and IIB (Choi et al., 2014) were increased by Mstn mutation (Lee et al., 2020). Therefore, Mstn mutant quail can serve as a proper avian model to investigate effects of Mstn mutation on myofiber types and meat quality in poultry. In the current study, we compared histochemical and meat quality characteristics of *pectoralis major* (PM) muscles between WT and Mstn mutant quail.

## 2 Materials and methods

### 2.1 Animals care

Japanese quail (*Coturnix japonica*) with a Mstn mutation were produced in our previous study (Lee et al., 2020). All animals used in this study were raised at the poultry facility at the Ohio State University (OSU) in Columbus, Ohio with the same environmental conditions such as consistent room temperature, the same brooder dimensions, and with free access to feed and water after hatch. All experimental procedures and animal care protocols were approved by the Institutional Animal Care and Use Committee (IACUC) of OSU (Protocol 2019A00000024).

### 2.2 Generation of Mstn mutant quail

As a previously reported (Lee et al., 2020), to analyze genotypes, the genomic DNA was extracted from feather germs and then targeted region in the *Mstn* gene was amplified by PCR with a specific primer set (F: 5′-GCA​TGG​ACG​AGC​TGT​ACA​AGT​A, R: 5′-CCC​TGC​TAA​TGT​TAG​GTG​CTT) at the condition followed by 35 cycles of 95°C for 40 s, 53°C for 40 s, 68°C for 30 s. The PCR product was sequenced at The Ohio State University Comprehensive Cancer Center.

### 2.3 Collection of muscle samples

To sample PM muscles, the male quail from the Mstn homozygous mutant (HO, n = 10) and WT (n = 12) lines were euthanized at 2 months of age by CO_2_ inhalation according to the IACUC protocol. Body weight (BW) and PM muscle weight (PMW) were measured, and breast percentage was calculated. After measurement of weights of whole PM, cross-sectional area (CSA) of the left PM muscle was measured in an area cut from the lower left to the upper right at the 1/2 point of the muscle (Scheuermann et al., 2004; Choi et al., 2014). Simultaneously, muscle samples (0.5 × 0.5 × 1.0 cm) from the left PM muscle were immediately frozen in liquid nitrogen and stored at −80°C for histochemical analysis. At 15 min *postmortem*, muscle pH value (pH_15 min_) was measured on each right muscle, and muscle samples were then immediately cooled with an ice-water slurry and stored at 4°C until meat quality analysis. After 24 h *postmortem*, meat quality characteristics, including pH_24 h_, meat color, drip loss, and cooking loss, were measured using the remaining left-side and entire right-side breasts.

### 2.4 Meat quality characteristics

Muscle pH values (pH_15 min_ and pH_24 h_) at the cranial region of the PM for each sample were measured using a Testo 206-pH2 (Testo AG, Lenzkirch, Germany) with a penetration probe. After 30 min of blooming time at 4°C, surface color of muscle samples at 24 h *postmortem* was determined using a spectrophotometer (CM-700d, Konica Minolta Inc., Ramsey, NJ). Color values, including lightness (*L* ^*^), redness (*a* ^*^), and yellowness (*b* ^*^), were assessed according to the recommendations of the Commission Internationale de l’Eclairage (1978). Drip loss was determined using a meat extract collector tube (Sarstedt Inc., Newton, NC), and percentages of drip loss were calculated with the difference in sample weight before and after 48 h at 4°C. For cooking loss, samples were weighed and put into a polyethylene bag, and then heated in a temperature-controlled water bath at 80°C until the core internal temperature reached 71°C (Honikel, 1998). Cooked samples were cooled in an ice-slurry until equilibration, and cooking loss percentage was calculated by weighing the samples before and after cooking.

### 2.5 Histochemical analysis

Serial muscle cross-sections (10 μm thickness) were obtained using a cryostat (CM1510S, Leica, Wetzlar, Germany) set at −25°C. To measure fiber characteristics, muscle sections were stained using the myosin ATPase staining kit (KTATP, StatLab, McKinney, TX) following the manufacturer’s instructions. All stained samples were analyzed using Image-Pro Plus software (Meida Cybernetics, Silver Spring, MD). In deep and superficial regions, more than 600 fibers in each region were used for statistical analysis of histochemical characteristics, such as percentages of the fiber type. The number percentage of each fiber type was calculated as the proportion of each of the fiber type numbers measured divided by the total fiber numbers measured.

### 2.6 Statistical analysis

BW, carcass, meat quality, and muscle fiber characteristics between the WT and HO quail lines were analyzed using a general linear mixed model procedure (SAS Institute, Cary, NC). Significant differences of the investigated parameters between the lines were evaluated using the probability difference by setting the significance level at 5% (*p* < 0.05). All data are presented as the least-squares means with standard errors.

## 3 Results

### 3.1 Increased body weight and breast muscle weight by myostatin mutation

The HO male quail exhibited a greater BW compared to the WT male quail at 2 months of age (115.7 g vs. 106.2 g, *p* < 0.001, Table 1). PMW, percentages of PMW, and CSA of PM muscle were approximately 18% (18.0 g vs. 15.2 g, *p* < 0.001), 9% (15.6% vs. 14.3%, *p* < 0.01), and 38% (251.4 mm^2^ vs. 182.0 mm^2^, *p* < 0.05) greater in the HO line than in the WT line, respectively (Table 1).

**TABLE 1:** Comparison of body weight and carcass traits between the wild type (WT) and myostatin homozygous mutant (HO) quail lines at 2-month of age.

	WT (n = 12)	HO (n = 10)	Level of significance
Body weight (g)	106.2 (1.39)^a^	115.7 (1.53)	***
PM muscle weight (g)	15.2 (0.34)	18.0 (0.38)	***
Breast percentage (%)	14.3 (0.22)	15.6 (0.23)	**
CSA of PM muscle (mm^2^)	236.0 (5.35)	295.7 (7.19)	***

Levels of significance: NS, no significant; **p < 0.01; ***p < 0.001.

^a^ Standard error of least-square means.

Abbreviations: PM, *pectoralis major*; CSA, cross-sectional area.

### 3.2 Effect of Mstn mutation on meat quality characteristics

As an indicator of glycolytic rate, the early *postmortem* muscle pH and ultimate pH values were not different between the HO and WT groups (Table 2). However, lightness values were greater in the HO quail line compared to the WT (54.3 vs. 52.6, *p* < 0.05); whereas no differences were observed in redness and yellowness values between the two groups (Table 2). The WT and Mstn mutant quail lines exhibited comparable percentage of drip loss (0.93% vs. 0.83%, *p* > 0.05). However, a higher cooking loss was observed in the Mstn mutant quail compared to the WT quail (8.56% vs. 6.06%, *p* < 0.05).

**TABLE 2:** Comparison of meat quality characteristics between the wild type (WT) and myostatin homozygous mutant (HO) quail lines at 2-month of age.

	WT	HO	Level of significance
pH_15 min_	6.34 (0.06)^a^	6.34 (0.07)	NS
pH_24 h_	5.84 (0.04)	5.91 (0.05)	NS
Lightness (L^*^)	52.6 (0.44)	54.3 (0.48)	*
Redness (a^*^)	6.70 (0.51)	6.43 (0.56)	NS
Yellowness (b^*^)	11.6 (1.99)	10.8 (2.18)	NS
Drip loss (%)	0.83 (0.07)	0.93 (0.07)	NS
Cooking loss (%)	6.06 (0.62)	8.56 (0.68)	*

Levels of significance: NS, no significant; *p < 0.05.

^a^ Standard error of least-square means.

### 3.3 Increased type IIB myofibers in the deep region of breast muscle by Mstn mutation

**FIGURE 1:**
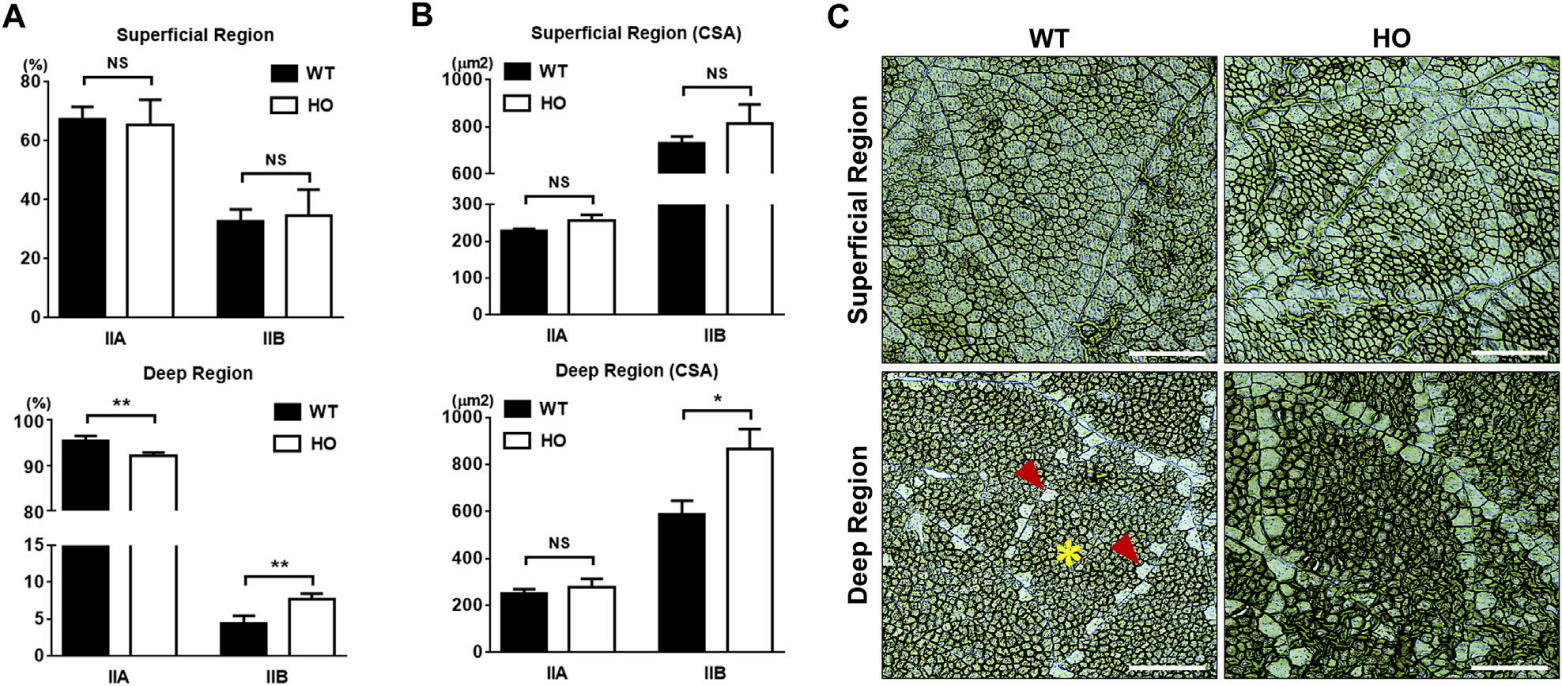
Comparison of muscle fiber composition and muscle fiber cross-sections in *pectoralis major* muscle between the wild type (WT) and myostatin homozygous mutant (HO) quail lines at 2-month of age. **(A)**. The percentages of type IIA and IIB muscle fibers in superficial or deep regions. **(B)**. The cross-sectional areas (CSA) of type IIA and IIB muscle fibers in superficial or deep regions. **(C)**. The representative images of the myofiber types of PM. Muscle fibers were stained using the myosin ATPase staining kit according to the manufacturer’s instructions, and type IIA muscle fibers stained darker than type IIB muscle fibers. Bars in graphs indicate standard errors. Level of significance: NS, no significant; *, *p* < 0.05; **, *p* < 0.01. Scale bars: 100 μm. Asterisk indicates type IIA myofibers and arrowheads are type IIB myofibers.

There were no significant differences in the percentages of type IIA (67.4% vs. 65.3%, *p* > 0.05, Figure 1A) and type IIB (32.6% vs. 34.7%, *p* > 0.05, Figure 1A) muscle fibers and CSA of the two types of muscle fibers (226.86 μm^2^ vs. 255.25 μm^2^, *p* > 0.05 for type IIA, and 729.25 μm^2^ vs. 814.43 μm^2^, *p* > 0.05 for type IIB, Figure 1B) in the superficial region between the WT and HO groups. However, the deep region in the HO group had lower percentages of numbers of type IIA muscle fiber compared to those in the WT group (92.2% vs. 95.6%, *p* < 0.01, Figure 1A). Conversely, higher percentages of type IIB muscle fiber numbers in deep regions were observed in the HO line than in the WT line (7.78% vs. 4.45%, *p* < 0.01, Figure 1A). For the CSA in the deep region, although there were no significant differences in the type IIA 251.29 μm^2^ vs. 278.27 μm^2^, *p* > 0.05, Figure 1B) between the WT and HO groups, the significance was shown in the type IIB (586.16 μm^2^ vs. 864.21 μm^2^, *p* < 0.05, Figure 1B) in the two groups. There was a difference in the composition of both muscle fiber types within the deep regions but not in the superficial regions between the WT and HO quail lines. The representative images of the myofiber types of PM are shown in Figure 1C.

## 4 Discussion

Mstn is a negative regulator in muscle growth and development, and is expressed almost exclusively in mature skeletal muscle (McPherron et al., 1997). The inhibitory role of Mstn in muscle development was further confirmed in numerous species of domesticated animals with double muscled phenotypes, including sheep, chicken, mice, cattle, and pigs (McPherron et al., 1997; McPherron and Lee, 1997; Kijas et al., 2007; Stinckens et al., 2008; Kim et al., 2022). Similar to our previous study (Lee et al., 2020), Mstn knock-out quail exhibited significantly heavier BW and greater breast muscle mass than WT quail, suggesting conserved function of Mstn in regulation of muscle growth between mammals and avian species.

Excessively increased muscle mass can develop into muscular abnormalities due to disrupted structure and functions of muscles which affect the rate and extent of *postmortem* metabolism and meat quality variation (Petracci et al., 2017). Pale, soft, and exudative (PSE)-like features in breast muscle have been an issue in fast-growing broilers (Barbut et al., 2008; Petracci et al., 2017). Generally, PSE-like conditions are characterized by low pH (<5.7) and high lightness values (>53) at 24 h *postmortem* in poultry species (Carvalho et al., 2014; Lee and Choi, 2021). In the present study, there were no significant differences in most of the meat quality indexes including *postmortem* pH, redness, yellowness, and drip loss of PM, except for a subtle decrease (1.5%) in cooking loss and an increased lightness value (1.7) in the mutant quail. However, the meat quality indexes were not confirmed with sensory testing since consumption of meat products from genome-edited animals has not yet been approved. It is still questionable whether the minor changes in meat quality characteristics in Mstn mutant quail can affect consumer satisfaction of meat products.

The architecture of the PM muscle from volant species has characteristics of locomotory muscles most specialized to produce power, and demonstrates an increasing proportion of slow-twitch muscle fibers along a ventral to dorsal area gradient (Rosser et al., 1987). In small birds, including quail, the deep regions composed primarily of type IIA muscle fibers are frequently activated for isometric function and sustained locomotory activity associated with flapping and flight (Rosser et al., 1987). The superficial regions with more glycolytic capacity show bursts of maximum power output through a series of very rapid and powerful contractions compared with the deeper areas (Rosser et al., 1987). In our previous study, breast muscles of quail, a volant species, consist mainly of type IIA and IIB muscle fibers due to their flight behavior, and type IIB muscle fibers were more abundantly found in the superficial regions of PM muscles compared to the deep regions (Choi et al., 2014). Fast-twitch muscle fibers, especially type IIB muscle fibers, are faster contracting muscle fibers with higher glycolytic capacity compared to slow-twitch muscle fibers (type I and IIA fibers) (Choi and Kim, 2009). It was reported that muscles lacking Mstn have faster and more glycolytic characteristics due to the myogenic transition from slow-twitch to fast-twitch muscle fibers (Qian et al., 2015; Baati et al., 2017). In chickens having only type IIB fibers in PM, Mstn mutation did not affect breast muscle weight, but increased leg muscle containing various fiber types (Kim et al., 2020). However, Mstn mutation in quail increased weights of breast muscle containing type IIA and IIB myofibers. This suggests degrees of muscle growth in response to Mstn mutation could vary depending on myofiber composition in muscle in avian species.

In general, muscles having a higher amount of type IIB muscle fibers can show higher glycolytic potentials with lower pH during the *postmortem* period compared to muscles having a lower amount of type IIB muscle fibers, leading to deterioration in meat quality of chicken (Lee and Choi, 2021). Mstn mutation in quail did not affect fiber types in superficial regions of PM, but slightly increased (approximately 3.3%) type IIB fibers in the deep region, possibly resulting in no difference in *postmortem* pH between the WT and Mstn mutant quail lines. These findings suggest that Mstn can be a candidate gene for increasing meat production without affecting meat quality in the poultry species.

## Data Availability

The raw data supporting the conclusion of this article will be made available by the authors, without undue reservation.
